# In Vivo Expression of Chicken Gut Anaerobes Identifies Carbohydrate- or Amino Acid-Utilising, Motile or Type VI Secretion System-Expressing Bacteria

**DOI:** 10.3390/ijms25126505

**Published:** 2024-06-13

**Authors:** Jana Rajova, Michal Zeman, Zuzana Seidlerova, Lenka Vlasatikova, Jitka Matiasovicova, Alena Sebkova, Marcela Faldynova, Hana Prikrylova, Daniela Karasova, Magdalena Crhanova, Pavel Kulich, Vladimir Babak, Jiri Volf, Ivan Rychlik

**Affiliations:** Veterinary Research Institute, CZ6210 Brno, Czech Republic; rajova@vri.cz (J.R.); michal.zeman.ext@geneton.sk (M.Z.); seidlerova@vri.cz (Z.S.); vlasatikova@vri.cz (L.V.); matiasovicova@vri.cz (J.M.); asebkova@vri.cz (A.S.); faldynova@vri.cz (M.F.); prikrylova@vri.cz (H.P.); karasova@vri.cz (D.K.); crhanova@vri.cz (M.C.); kulich@vri.cz (P.K.); babak@vri.cz (V.B.); volf@vri.cz (J.V.)

**Keywords:** chicken microbiota, caecum, gene expression, mass spectrometry, anaerobe, metabolism

## Abstract

Complex gut microbiota increases chickens’ resistance to enteric pathogens. However, the principles of this phenomenon are not understood in detail. One of the possibilities for how to decipher the role of gut microbiota in chickens’ resistance to enteric pathogens is to systematically characterise the gene expression of individual gut microbiota members colonising the chicken caecum. To reach this aim, newly hatched chicks were inoculated with bacterial species whose whole genomic sequence was known. Total protein purified from the chicken caecum was analysed by mass spectrometry, and the obtained spectra were searched against strain-specific protein databases generated from known genomic sequences. *Campylobacter jejuni*, *Phascolarctobacterium* sp. and *Sutterella massiliensis* did not utilise carbohydrates when colonising the chicken caecum. On the other hand, *Bacteroides*, *Mediterranea*, *Marseilla*, *Megamonas*, *Megasphaera*, *Bifidobacterium*, *Blautia*, *Escherichia coli* and *Succinatimonas* fermented carbohydrates. *C. jejuni* was the only motile bacterium, and *Bacteroides mediterraneensis* expressed the type VI secretion system. Classification of in vivo expression is key for understanding the role of individual species in complex microbial populations colonising the intestinal tract. Knowledge of the expression of motility, the type VI secretion system, and preference for carbohydrate or amino acid fermentation is important for the selection of bacteria for defined competitive exclusion products.

## 1. Introduction

With roughly 25 billion chickens bred in 2020, chickens numerically represent the most widespread farm animal in the world [1]. The vast majority of chickens are kept in intensive production systems that start in hatcheries where chicks are hatched from cleaned and disinfected eggs, without any contact with the parent birds. However, it is known that contact between chicks and adult birds is important for transfer of chicken-adapted gut microbiota [2], which, in turn, increases the chick’s resistance to enteric diseases [3,4,5]. Well-developed gut microbiota is also linked to other positive benefits for the chicken host such that the composition and function of the whole community, as well as of its individual members, are of considerable importance.

The function and metabolic potential of bacterial species colonising the chicken intestinal tract can be predicted from their genomic sequence, obtained either by sequencing of DNA from pure bacterial cultures [6] or by metagenomic sequencing of DNA purified from complex samples [7,8]. In fact, there are protocols that allow prediction of the metabolic potential of a given community based on its microbial composition [9]. However, not a single viable organism expresses all the genes it encodes simultaneously. Instead, only a subset of genes is always expressed, and among the expressed genes, expression levels may differ by orders of magnitude. Analysis of the genomic sequence is therefore only the first step in predicting the biological role of a particular bacterium in a given environment, and additional tools must be applied if more precise information is needed. Additional information on gene expression can be obtained by RNA sequencing or by protein mass spectrometry [10]. Both RNA sequencing and protein mass spectrometry rely on nucleic acid or amino acid sequence data, which are usually extracted from public databases. The obtained information is thus influenced by the sequences in the databases and the strains present in the sample [10,11]. Since this always contains an unknown amount of uncertainty, the cumulation of these uncertainties may lead to mistaken association of some of the expressed genes with particular strains due to sequence similarities, or, conversely, expression of some of the genes may remain undetected due to their absence from public databases. As a consequence, there are limited data on the precise gene expression of particular bacterial species when they are colonising the intestinal tract despite the fact that such information is essential for understanding the function of individual gut microbiota members and for rational selection of future probiotic strains.

We have recently presented data on whole-genome sequencing of bacterial species from the chicken intestinal tract [6]. Later on, we showed that only some of the bacterial isolates efficiently colonised the caecum of newly hatched chicks after single-dose administration [12]. In this study, we combined these data to determine protein expression in selected species when colonising the chicken caecum. Known whole genomic sequences were used for the construction of strain-specific protein databases for protein mass spectrometry. By orally inoculating chicks with strains that are usually absent from the microbiota of one-week-old chicks but that efficiently colonise the chicken caecum [12], we were able to determine the in vivo protein expression of the introduced bacterial strain. This protocol was applied in 20 different strains and used to differentiate between carbohydrate-dependent and independent gut colonisers and to define butyrate, acetate, succinate and formate producers as well as motile gut colonisers and isolates expressing the type VI secretion system in vivo. Such information helps clarify the function of individual chicken caecal microbiota members and allows the selection of appropriate bacterial species into novel competitive exclusion products [13].

## 2. Results

### 2.1. Bacterial Colonisation

**Figure 1:**
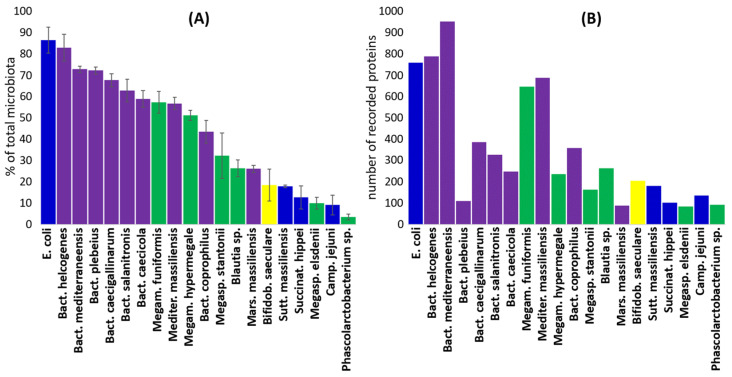
Abundance of strains in the caecum as determined by 16S rRNA gene sequencing. The used bacterial strains efficiently colonised the chicken caecum (Panel (**A**)). Eighty-three (*M. elsdenii*) to nine hundred fifty-two (*B. mediterraneensis*) different proteins were recorded as expressed in vivo for individual strains (Panel (**B**)). Blue—Proteobacteria, purple—Bacteroidetes, green—Firmicutes, yellow—Actinobacteria.

Sequencing the V3/V4 variable region of 16S rRNA genes was used only to check the efficiency of colonisation by target strains, and we therefore did not develop this analysis further (see Table S1 for all OTUs detected in this study). *Escherichia coli* and *Bacteroides helcogenes* formed around 85% of total caecal microbiota, while the least abundant strains included *Megasphaera elsdenii*, *Campylobacter jejuni* and *Phascolarctobacterium* sp., forming 3–10% of total microbiota (Figure 1A).

**Figure 2:**
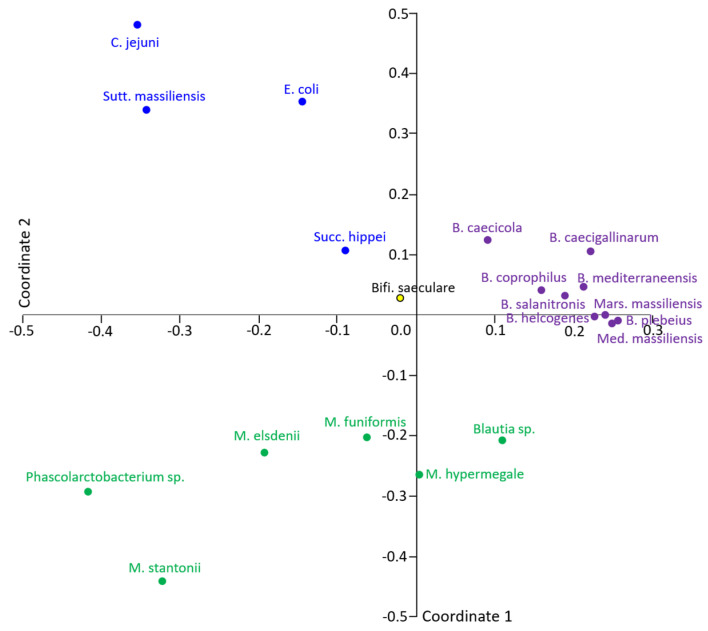
Clustering of the strains based on in vivo expression of major proteins. The PCoA clustering using Raup–Crick distances corresponded to the taxonomic classification of individual strains, showing that related strains expressed similar proteins. Blue—Proteobacteria, purple—Bacteroidetes, green—Firmicutes, yellow—Actinobacteria.

More than 700 proteins were expressed in vivo in the most abundant strains. On the other hand, *M. elsdenii*, with 83 identified proteins, defined the lowest inclusion threshold (Figure 1B). Comparison of in vivo expression performed over the 25 most abundant proteins of each strain showed that Bacteroidetes isolates clustered close to each other, and clustering of other strains also corresponded to their taxonomic classification (Figure 2).

### 2.2. Commonly Expressed Proteins without Enzymatic Function

**Figure 3:**
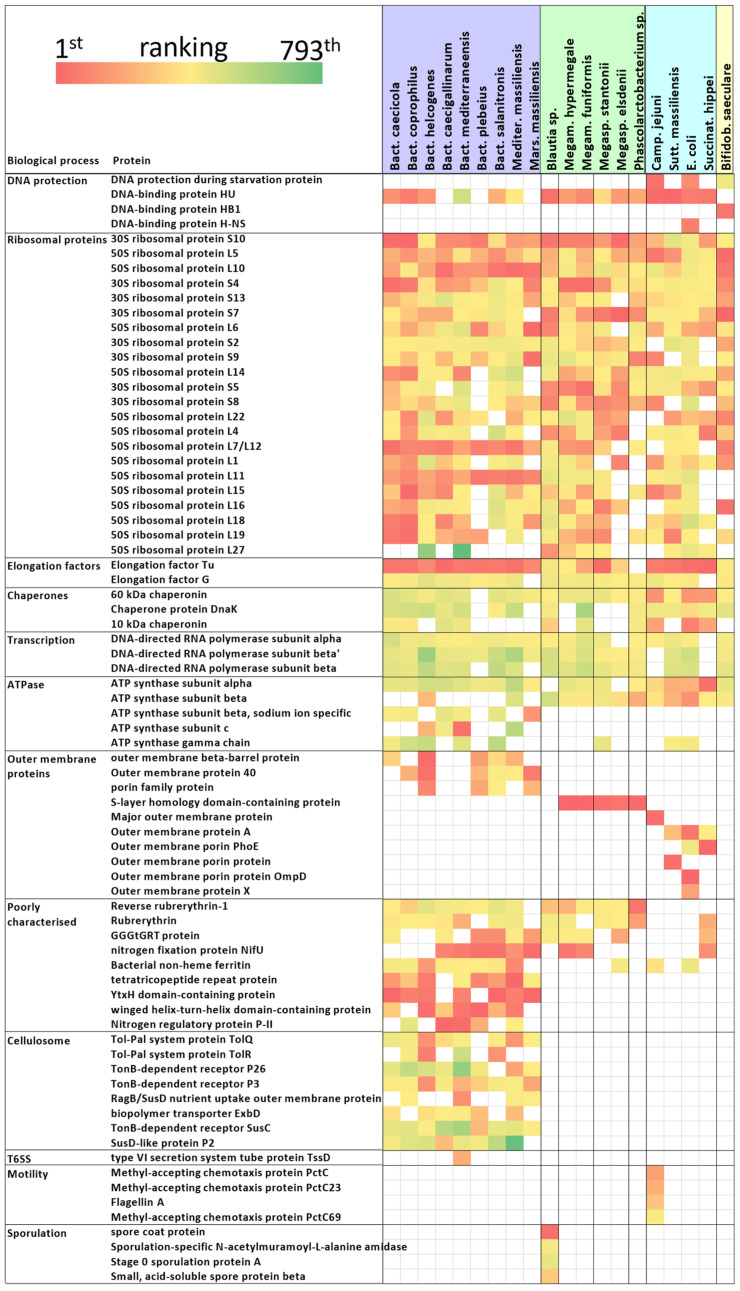
In vivo expression of proteins without enzymatic function. The heat map was generated using ranking classification of the most expressed proteins without enzymatic function or with unknown function. Red colour means the top ranking and therefore the highest expression. Yellow indicates moderate expression, and shades of green are used for low expression of proteins. The number of different ribosomal proteins was reduced to fit the whole figure on one page. For a full list of detected proteins, please see Table S2. Background colours of bacterial taxa: purple—Bacteroidetes, green—Firmicutes, blue—Proteobacteria, yellow—Actinobacteria.

Ribosomal proteins formed numerically the most abundant group of proteins, and ribosomal proteins S4, S10, L5 and L10 were recorded as expressed in vivo by all 20 strains. Elongation factor Tu was expressed and detected in 19 strains and DNA-binding HU protein in 16 strains, respectively (Figure 3). Additional highly expressed proteins without enzymatic function were restricted only to particular taxa. Outer membrane proteins were expressed by all Gram-negative Bacteroidetes and Proteobacteria. All strains from the order Selenomonadales (*Megamonas*, *Megasphaera* and *Phascolarctobacterium*) expressed S-layer homology domain-containing proteins [14,15]. These proteins ranked among the top five proteins in these genera, and *Megasphaera* and *Phascolarctobacterium* expressed multiple variants of this type of protein. Rubrerythrin and reverse rubrerythrin-1 were expressed in Bacteroidetes, Firmicutes and *Succinatimonas*. Poorly characterised GGGtGRT protein was expressed in Bacteroidetes, *Blautia*, *Megamonas*, *Megasphaera* and *Succinatimonas*. The nitrogen fixation protein NifU was expressed in Bacteroidetes, *Megamonas* and *Succinatimonas*. Tetratricopeptide repeat protein, YtxH domain-containing protein, winged helix-turn-helix domain-containing protein, nitrogen regulatory protein P-II, Tol–Pal system proteins, TonB-dependent receptor proteins and RagB/SusD family nutrient uptake outer membrane proteins were expressed exclusively in Bacteroidetes [11] (Figure 3). *Bacteroides mediterraneensis* expressed the TssD tube protein of the type VI secretion system [16]. *Campylobacter jejuni* was the only motile bacterium in the caecum of those tested, as 3 different methyl-accepting chemotaxis proteins and flagellin A were recorded as expressed. Many *Blautia* cells entered sporulation or were present in the form of spores in the caecum, as spore coat protein was the third most abundant protein of *Blautia* sp., and stage 0 sporulation protein A, sporulation-specific N-acetylmuramoyl-L-alanine amidase and small, acid-soluble spore protein beta of *Blautia* sp. were detected as expressed as well (Figure 3).

### 2.3. Glycolytic Enzymes

**Figure 4:**
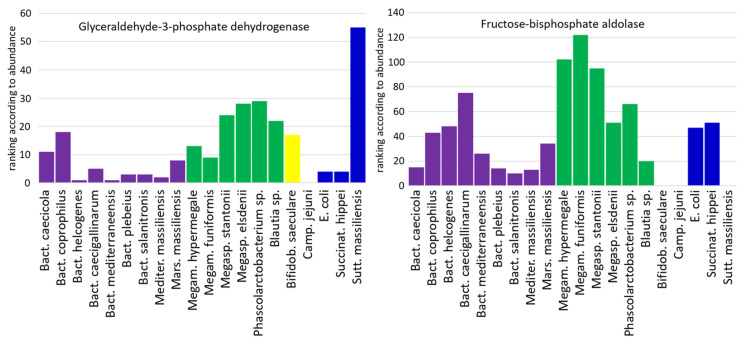
Expression of GAPDH and fructose-bisphosphate aldolase by chicken gut anaerobes in vivo. Bacteroidetes, *Megamonas*, *Megasphaera*, *Blautia*, *Bifidobacterium*, *E. coli* and *Succinatimonas* were dependent on carbohydrate metabolism, while *C. jejuni* and *Sutterella* carried out metabolism that was nearly independent of carbohydrate degradation. Purple—Bacteroidetes, green—Firmicutes, yellow—Actinobacteria, blue—Proteobacteria.

Glycolytic enzymes ranked among the most abundant proteins in most, but not all, of the strains. *C. jejuni* colonised the chicken caecum without expressing any glycolytic enzymes. Glyceraldehyde-3-phosphate dehydrogenase (GAPDH) was the only glycolytic enzyme expressed in *Sutterella*, ranking as the 55th most abundant protein in that bacterium. *Phascolarctobacterium* sp. was the last species that did not preferentially utilise carbohydrates, since only fructose-bisphosphate aldolase and GAPDH were expressed as the 29th and 66th most abundant proteins, respectively (Figure 4). On the other hand, all Bacteroidetes and both *Megamonas* and *Megasphaera* as well as *Blautia*, *E. coli* and *Succinatimonas* expressed most glycolytic enzymes, while *Bifidobacterium* fermented carbohydrates by the *Bifidobacterium* shunt, characterised by high expression of transaldolase, transketolase and xylulose-5-phosphate/fructose-6-phosphate phosphoketolase. GAPDH was expressed the most in Bacteroidetes (GAPDH was the most abundant protein in *B. helcogenes* and *B. mediterraneensis*), *E. coli* and *Succinatimonas*. GAPDH was of lower importance for *Megamonas* species, *Megasphaera* species, *Blautia* and *Bifidobacterium*. The second most expressed enzyme from glycolysis, i.e., fructose-bisphosphate aldolase, confirmed that glycolysis was central for Bacteroidetes, *E. coli* and *Succinatimonas* and was less important for *Megamonas* and *Megasphaera* (Figure 4).

### 2.4. Additional Broadly Expressed Proteins with Enzymatic Function

**Figure 5:**
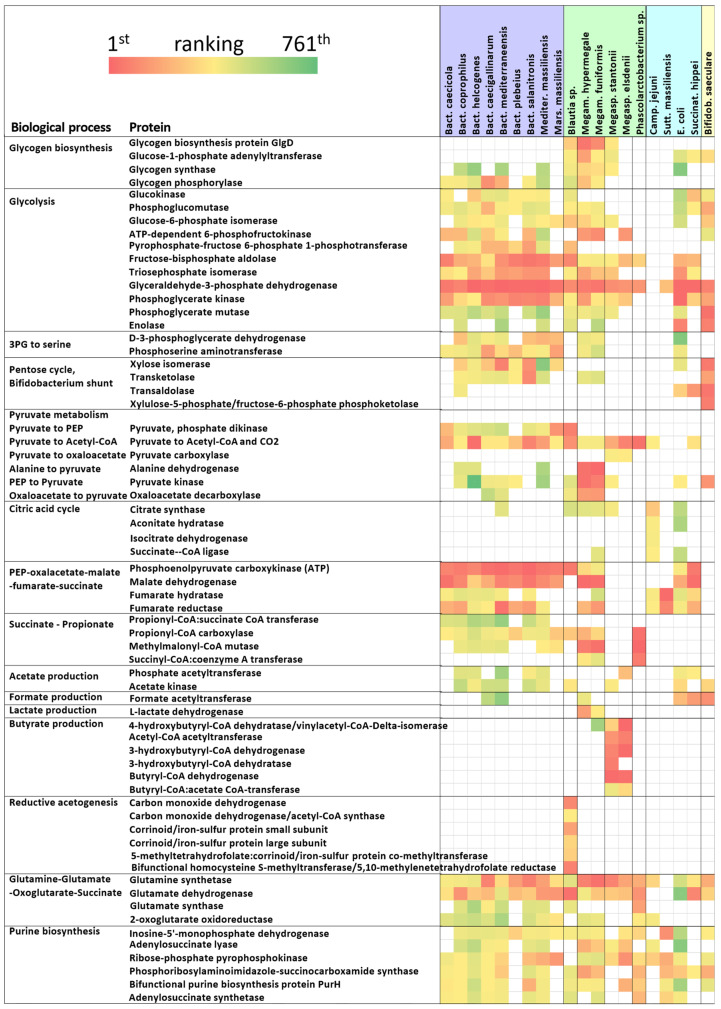
In vivo expression of proteins with assigned enzymatic function. The heat map was generated using ranking classification. Red colour means the top ranking and therefore the highest expression. Yellow colour indicates moderate expression, and shades of green are used for low expression of proteins. The most abundant enzymes are listed in this figure. For a full list of all detected proteins, please see Table S2. Background colours of bacterial taxa: purple—Bacteroidetes, green—Firmicutes, blue—Proteobacteria, yellow—Actinobacteria.

Glutamine synthetase and glutamate dehydrogenase were expressed in 19 and 18 strains, respectively. Neither of these enzymes was expressed by *Sutterella massiliensis*. Inosine-5′-monophosphate dehydrogenase, involved in biosynthesis of purines, was expressed in 16 strains out of 20 tested. At least one subunit (alpha or beta) of ATP synthase was expressed by all tested bacterial species. The beta subunit was not detected in *B. plebeius*, and the alpha subunit was not detected in *M. elsdenii* or in *Blautia* sp. In the remaining species, both subunits of ATP synthase were expressed. All representatives of Proteobacteria (*C. jejuni*, *E. coli*, *Succ. hippei* and *Sutt. massiliensis*) were highly dependent on ATP production by ATP synthase, since both its subunits ranked among top 40 expressed proteins. *Bacteroides* differed from the remaining species, since, in six out of nine species, the sodium ion specific beta subunit of ATP synthase was expressed (Figure 5).

### 2.5. In Vivo Metabolism in Individual Gut Microbiota Members

The remaining enzymatic pathways expressed by chicken gut anaerobes are presented, for clarity, according to their taxonomic classification.

The most active process of *C. jejuni* was sensing and controlling the redox status of its cytoplasm. Alkyl hydroperoxide reductase C was the most expressed protein, and thiol peroxidase ranked as the eighth most expressed protein. In addition, catalase, thioredoxin reductase and superoxide dismutase all belonged among the top 100 expressed proteins. *C. jejuni* was active in one-carbon metabolism using S-adenosylmethionine synthase, 5-methyltetrahydropteroyltriglutamate-homocysteine methyltransferase and serine hydroxymethyltransferase, and utilised hydrogen via quinone-reactive Ni/Fe-hydrogenase. *C. jejuni* was the only species expressing TCA cycle proteins, since, except for malate dehydrogenase, all the TCA enzymes were recorded as expressed in vivo in that species, including citrate synthase, aconitate hydratase, isocitrate dehydrogenase, 2-oxoglutarate oxidoreductase, succinate-CoA ligase, fumarate reductase and fumarate hydratase. Input acetyl-CoA was generated from pyruvate by pyruvate:ferredoxin oxidoreductase (Figure 5).

Reduced NAD and FAD were anaerobically respired via formate dehydrogenase, trimethylamine-N-oxide reductase, nitrate reductase or 5-hydroxyisourate hydrolase. Amino acids served as a source of organic carbon, since periplasmic serine endoprotease DegP, ABC transporter glutamine-binding protein GlnH, putative histidine-binding protein, branched-chain-amino-acid aminotransferase and aspartate ammonia-lyase were among the highly expressed proteins of *C. jejuni* colonising the chicken caecum.

*Sutterella* utilised proteins and peptides as major carbon sources, since isoaspartyl dipeptidase, peptidase E, dipeptidase and carboxypeptidase G were among the highly expressed proteins. Asparagine and aspartate represented two central amino acids for *Sutterella*, since these could be produced by isoaspartyl dipeptidase, indole-3-acetyl-aspartic acid hydrolase or asparaginase. Aspartate was then converted to fumarate by aspartate ammonia-lyase, and fumarate was metabolised either to succinate via fumarate reductase or to malate via fumarate hydratase. The asparagine–aspartate–fumarate–malate pathway was central for *Sutterella*, since enzymes catalysing subsequent steps ranked sixth, seventh and fourth in protein abundance, respectively (Figure 5). Arginine was another amino acid important for *Sutterella*. Arginine was captured by highly expressed ABC transporter arginine-binding protein and converted to argininosuccinate and citrulline by argininosuccinate lyase and argininosuccinate synthase.

*Phascolarctobacterium* sp. preferred amino acids as a carbon source due to expression of L-cystine-binding protein, L-serine ammonia-lyase, glutaconyl-CoA decarboxylase and Leu/Ile/Val-binding protein. Propionyl-CoA carboxylase, methylmalonyl-CoA epimerase, methylmalonyl-CoA mutase and succinyl-CoA:coenzyme A transferase were among the top expressed enzymes allowing succinate production (Figure 5). The expression of acetyl-CoA:oxalate CoA transferase was specific to *Phascolarctobacterium* sp., but since we did not find expression of any other enzyme involved in oxalate metabolism, this finding will need to be independently confirmed.

Bacteroidetes represented the polysaccharide degraders in the chicken caecum. TonB-dependent receptor proteins and RagB/SusD nutrient uptake outer membrane proteins were mentioned above. Following polysaccharide degradation, monosaccharides were processed further through glycolysis down to 3-phospho-D-glycerate. The rate of glycolysis decreased at this step, since low or no expression of phosphoglycerate mutase and enolase was recorded in different Bacteroidetes isolates. Instead, high expression of D-3-phosphoglycerate dehydrogenase and phosphoserine aminotransferase was recorded; these enzymes convert 3-phospho-D-glycerate to serine (Figure 5).

Additional highly expressed proteins in Bacteroidetes included malate dehydrogenase and phosphoenolpyruvate carboxykinase (ATP). Together with moderately expressed fumarate hydratase, these enzymes allowed the conversion of phosphoenolpyruvate into fumarate. *Mediterranea* and all *Bacteroides* used fumarate as an electron acceptor in anaerobic respiration with fumarate reductase. Expression of fumarate reductase was not recorded in *Marseilla*, and this species expressed anaerobic nitric oxide reductase instead (Figure 5). Phosphoenolpyruvate was converted also to pyruvate by moderately expressed pyruvate kinase, and the resulting pyruvate was transformed into acetyl-CoA by pyruvate:ferredoxin oxidoreductase. Acetyl-CoA was converted to acetate by phosphate acetyltransferase and acetate kinase (Figure 5).

The last pathway expressed in Bacteroidetes, though expressed at a lower level than the previous two pathways, allowed the production of propionate from succinate via succinyl-CoA, methylmalonyl-CoA and propionyl-CoA. Moderate expression of propionyl-CoA:succinate CoA transferase was recorded only in Bacteroidetes, closing the cycle of succinate-to-propionate conversion.

Both *Megamonas* species expressed similar glycolytic enzymes to Bacteroidetes, including D-3-phosphoglycerate dehydrogenase and phosphoserine aminotransferase, diverting 3-phospho-D-glycerate from glycolytic degradation towards serine biosynthesis. Otherwise, pyruvate was the most characteristic molecule for *Megamonas*. Alanine dehydrogenase ranked as the seventh and second most expressed protein in *M. hypermegale* and *M. funiformis*, respectively, allowing pyruvate production from alanine. Pyruvate could also be produced also from lactate by lactate dehydrogenase, from phosphoenolpyruvate by pyruvate kinase and from oxaloacetate by oxaloacetate decarboxylase. All these enzymes were highly expressed in both *Megamonas* species. Interconversion between phosphoenolpyruvate and oxaloacetate was also possible in *Megamonas* due to the expression of phosphoenolpyruvate carboxykinase. Oxaloacetate could be transformed to malate, fumarate and succinate. *Megamonas* likely accumulated glycogen, since both species expressed glycogen biosynthesis protein GlgD, glycogen synthase, glucose-1-phosphate adenylyltransferase and glycogen operon protein GlgX, all regulating glycogen biosynthesis, as well as glycogen phosphorylase, enabling glycogen degradation (Figure 5).

Carbohydrate metabolism in *Megamonas* was complemented by amino acid fermentation. *Megamonas* expressed leucine-, isoleucine-, valine-, threonine- and alanine-binding protein and high-affinity branched-chain amino acid transport ATP-binding protein LivF. Branched amino acids were fermented to propionyl-CoA, which was converted to acetate or succinate via methylmalonyl-CoA–succinyl-CoA isomerisation [11]. Succinyl-CoA could be converted to succinate by the activity of succinyl-CoA:coenzyme A transferase, with parallel conversion of acetate to acetyl-CoA.

Both *Megasphaera* species expressed the glycolytic pathway down to pyruvate. Pyruvate could be transformed to oxaloacetate by pyruvate carboxylase, though the majority of pyruvate was converted to acetyl-CoA. Acetyl-CoA was used for butyrate production, since two molecules of acetyl-CoA were fused to acetoacetyl-CoA by acetyl-CoA acetyltransferase followed by enzymatic activity of 3-hydroxybutyryl-CoA dehydrogenase, 3-hydroxybutyryl-CoA dehydratase, butyryl-CoA dehydrogenase and butyryl-CoA:acetate CoA-transferase. Acetate and acetyl-CoA could originate from glycolysis, but *Megasphaera* also expressed lactate utilisation proteins, implying that lactate could be used for acetyl-CoA production as well. Both *Megasphaera* species were also able to produce butyrate from 4-hydroxybutyrate, since 4-hydroxybutyryl-CoA dehydratase was expressed by *Megasphaera* in vivo as well (Figure 5).

*Succinatimonas* preferentially degraded carbohydrates, which were imported to the cytoplasm by galactofuranose ABC transporter periplasmic-binding protein YtfQ and ribose import binding protein RbsB and further modified by fucose isomerase. Following glycolysis, pyruvate was converted into formate and acetyl-CoA by formate acetyltransferase. *Succinatimonas* also utilised fumarate, which could be produced from phosphoenolpyruvate via oxalacetate and malate and reduced to succinate (Figure 5).

*E. coli* used glycolysis when colonising the chicken caecum. All glycolytic enzymes were expressed down to formate acetyltransferase, phosphate acetyltransferase and acetate kinase, resulting in production of formate and acetate (Figure 5). The preference for carbohydrate metabolism was further supported by the expression of carbohydrate transport, degradation and isomerisation proteins such as ribose import binding protein RbsB, D-galactose-binding periplasmic protein, L-arabinose-binding periplasmic protein, maltose/maltodextrin-binding periplasmic protein, maltoporin alpha-galactosidase, L-fucose isomerase, D-galactonate dehydratase and N-acetylneuraminate lyase. *E. coli* also expressed enzymes converting aspartate to fumarate, and phosphoenolpyruvate to oxaloacetate, malate and fumarate. Fumarate was then transformed to succinate, which was likely another end product of *E. coli* metabolism in the chicken caecum. *E. coli* also highly expressed lactaldehyde reductase and glycerol dehydrogenase.

*Blautia* combined glycolytic carbohydrate fermentation with reductive acetogenesis (Figure 5). *Blautia* expressed all glycolytic enzymes required for degradation of glucose to pyruvate. We also recorded high expression of phosphoenolpyruvate carboxykinase (ATP) and phosphate propanoyltransferase, which ranked as the 11th and 18th most expressed proteins, respectively. However, these enzymes were apparent orphans with no clear link to other steps in the metabolism of propionate or oxaloacetate.

Reductive acetogenesis, i.e., assimilation of CO_2_ into organic carbon in the form of acetate, was specific to *Blautia*. Enzymes required for this pathway were highly expressed, including carbon monoxide dehydrogenase 1, the alpha subunit of carbon monoxide dehydrogenase/acetyl-CoA synthase, the large and small subunits of corrinoid/iron–sulfur protein, 5-methyltetrahydrofolate:corrinoid/iron–sulfur protein co-methyltransferase and bifunctional homocysteine S-methyltransferase/5,10-methylenetetrahydrofolate reductase (Figure 5).

*Bifidobacterium* fermented carbohydrates by the *Bifidobacterium* shunt of glycolysis [17], since phosphoketolase, transaldolase, transketolase and xylose isomerase were among the highly expressed proteins. Acetyl-P, once formed, was converted to acetate by acetate kinase, and glyceraldehyde-3-P entered glycolysis, in which it was metabolised down to pyruvate. Pyruvate was finally transformed to formate and acetyl-CoA by formate acetyltransferase (Figure 5).

### 2.6. Confirmation of Predicted Phenotypes—Cellulosomes

**Figure 6:**
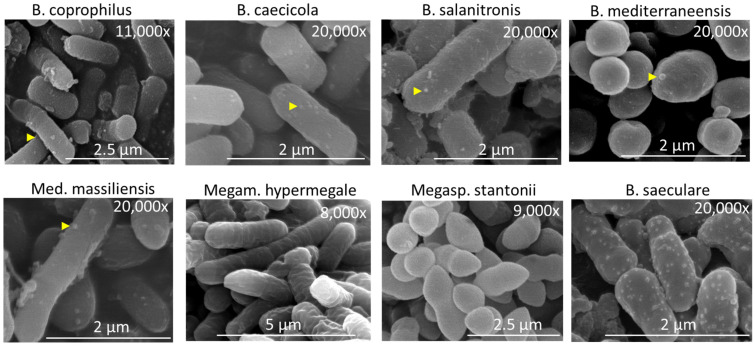
Cellulosomes on the surface of *Bacteroides* sp. and *Mediterranea*. In vitro-grown representatives of Bacteroidetes (*Bacteroides* and *Mediterranea*) together with control *M. hypermegale*, *M. stantonii* and *B. saeculare* were subjected to scanning electron microscopy. Surface structures similar to cellulosomes (highlighted with yellow arrowheads) were recorded in all *Bacteroides* and *Mediterranea*. Such structures were absent in *Megamonas* and *Megasphaera*, though similar surface structures were also observed in *B. saeculare*.

All *Bacteroides* species and *Mediterranea* expressed cellulosomes in vivo. Since genes for cellulosomes are common and highly expressed in *Bacteroides* [6,11], and since cellulosome structures can be observed by electron microscopy [18], we verified cellulosome expression in *Bacteroides* in the last experiment. Four *Bacteroides* species, *Mediterranea*, *M. hypermegale*, *M. stantonii* and *B. saeculare* grown in vitro were subjected to scanning electron microscopy. Surface structures similar to cellulosomes were recorded in all *Bacteroides* species and *Mediterranea* but were absent in *Megamonas* and *Megasphaera* (Figure 6). Similar surface structures were observed also in *B. saeculare*. Since *B. saeculare* is a Gram-positive bacterium without an outer membrane, the structures in *B*. *saeculare* must have represented a different molecular complex, as shown in *Clostridium thermocellum* [19].

## 3. Discussion

There are several limits to this study. Protein function was predicted by automatic annotation. To limit the consequences of error in automatic annotation, we primarily considered enzymatic pathways in which multiple enzymes were expressed. For the same reason, we did not discuss the expression of proteins that showed high levels of expression but without expression of other enzymes belonging to the same metabolic pathway. We also did not aim to describe differences in individual *Bacteroides*, *Megamonas* or *Megasphaera* species. Instead, the fact that similar proteins were expressed in different species of the same genus was used as an additional, though indirect, evidence of correctly identified metabolic pathways. Finally, there were many expressed hypothetical proteins and these may catalyse yet unknown enzymatic reactions or may catalyse known reactions by a yet unknown mechanism.

The second set of limitations is that the expression levels were arranged based on ranking according to PSM counts assuming that a higher amount of a particular protein means its higher importance. Such assumption need not be correct in all cases and enzymatic activity and substrate affinity, in addition to plain protein amount, may influence the final output. Similarly, structural and regulatory proteins can be present at a constant abundance, and their posttranslational modification may have dramatic consequences for their biological activities. Last critical point is that many enzymes can operate in both directions depending on availability of substrates. In this study we had to decide for the most likely direction of each reaction depending on the expression of all other enzymes in a given pathway.

Proteins involved in DNA binding, transcription or protein synthesis were similarly expressed in all the strains. Outer membrane proteins were recorded in Bacteroidetes and Proteobacteria, and these were replaced by S-layer homology domain-containing proteins in *Megamonas*, *Megasphaera* and *Phascolarctobacterium*. S-layer homology domain-containing proteins have not been detected in these genera to date, but related *Selenomonas* and *Mitsuokella* are known to express them [14,15]. Motility was recorded only in *C. jejuni*, showing that motility is not common for microbiota present in caecal digesta, as proposed before [6]. Spore formation in *Blautia* was expected. Unlike motility, spore formation is widespread in gut microbiota due to the presence of different species belonging to the order Clostridiales [6]. Efficient spore formation is also in agreement with the common appearance of Clostridiales, including *Blautia*, among the first anaerobes [20,21,22] colonising newly hatched chicks from the environment in the form of spores [23,24,25,26,27]. Commonly expressed rubrerythrins serve to control oxygen species [28,29]. GGGtGRT protein has been recorded only as conserved in genomes of taxonomically unrelated species thus far. Here we show that this protein is highly expressed in vivo by distantly related Gram-positive and Gram-negative taxa such as *Bacteroides*, *Megamonas*, *M. elsdenii*, *S. hippei* and *Blautia* sp. High expression was recorded also for tetratricopeptide repeat protein, YtxH domain-containing protein and winged helix-turn-helix domain-containing protein. Although these proteins were expressed only by Bacteroidetes, they ranked among the top 10 most abundant proteins, indicating an important function of these proteins for Bacteroidetes. Bacteroidetes also expressed Tol–Pal system proteins, TonB-dependent receptor proteins and RagB/SusD family nutrient uptake outer membrane proteins and formed cellulosomes on the surface of their cells [30]. In vivo expression of these proteins was also recorded in our previous study [11], and cellulosome structures were confirmed on the surface of *Bacteroides*. *B. mediterraneensis* expressed the type VI secretion system tube protein TssD. *Bacteroides* encode different T6SSs [16], and the presence of these systems enables them to outcompete similar species in vitro and in vivo [31,32]. Here, we provide direct evidence of T6SS expression in *Bacteroides* in vivo at a high level, similar to the expression of ribosomal proteins.

*C. jejuni* is known to utilise amino acids and simple organic acids as electron donors and carbon sources in the citrate cycle [33,34,35,36]. For both *C. jejuni* and *Phascolarctobacterium*, carbohydrate-independent metabolism in vivo was predicted previously [11]; however, *Sutterella* has not been associated with non-carbohydrate metabolism thus far. The remaining bacterial species fermented carbohydrates. The same glycolytic enzymes were expressed in all species except for *Bifidobacterium*, which expressed enzymes already reported as specific to *Bifidobacterium* when grown in vitro [37,38].

Three metabolic pathways were generally expressed among gut microbiota members. We have previously detected the propionate–propionyl-CoA–methylmalonyl-CoA–succinyl-CoA–succinate pathway as expressed by *Bacteroides*, *Megamonas* and *Phascolarctobacterium* [10], the direction of which is dependent on cobalamin availability [39]. Here, we expand on the original observations in the sense that this pathway leads towards propionate production in *Bacteroides* colonising the chicken caecum due to the expression of propionyl-CoA:succinate CoA transferase releasing free propionate from propionyl-CoA. In *Megamonas* and *Phascolarctobacterium*, this pathway likely operates in the opposite direction due to the expression of multiple branched amino acid binding proteins followed by degradation of such amino acids into propionyl-CoA entering this pathway [40].

*Megasphaera* was the only bacterial species expressing all enzymes required for butyrate production from acetyl-CoA. Predictions from genomic sequences indicated that *Megasphaera* might be capable not only of butyrate production but also of acetate, formate and caproate production [41]. Maki and Looft then detected production of acetate, propionate and isovalerate in vitro, in addition to butyrate [42]. This shows that predictions from genomic sequences may be of questionable value, since they place all potential end products at the same level, and in vitro experiments with an artificially increased concentration of a particular substrate may lead to correct results but conflicting meanings. When *Megasphaera* colonises the chicken caecum, the butyrate production pathway dominates its metabolism and is central to *Megasphaera* function in the gut.

Fumarate–succinate conversion is a known mode of recycling reduced NAD or FAD under anaerobic conditions [43,44]. Conversion of phosphoenolpyruvate to fumarate via oxaloacetate and malate may thus provide enough fumarate for anaerobic respiration, since malate dehydrogenase and phosphoenolpyruvate carboxykinase were expressed at the same level as the most abundant glycolytic enzymes in *Bacteroides*, *Megamonas* and *Succinatimonas*.

## 4. Materials and Methods

### 4.1. Ethical Approval

The handling of animals in the study was performed in accordance with current Czech legislation (Animal Protection and Welfare Act no. 246/1992 Collection of the Government of the Czech Republic). The specific experiments were approved by the Committee for Animal Welfare of the Ministry of Agriculture of the Czech Republic on 15 January 2018 (permit number MZe1922).

### 4.2. Chickens

The study was performed in accordance with Animal Research: Reporting of In Vivo Experiments (ARRIVE) guidelines (https://arriveguidelines.org/arrive-guidelines, accessed on 5 January 2024)). Male ISA Brown chicks were obtained from a local hatchery on the day of hatching. The chicks were housed in an air-conditioned animal house with a controlled light programme and ad libitum access to feed and drinking water. Chicks were inoculated orally on the day of hatching and sacrificed one week later. At day 8, chicks were euthanized by intravenous administration of 0.1 mL of T-61 (MSD Animal Health, Prague, Czech Republic) followed by decapitation and necropsy.

### 4.3. Bacterial Strains

Newly hatched chicks (3 chicks per bacterial strain) were orally inoculated with *Bacteroides caecigallinarum* An428b, *Bacteroides coprophilus* ET5, *Bacteroides helcogenes* ET71, *Bacteroides mediterraneensis* An793, *Bacteroides plebeius* ET8, *Bacteroides salanitronis* An322, *Bacteroides caecicola* ET2, *Mediterranea massiliensis* An502, *Marsiella massiliensis* ET9, *Megamonas hypermegale* An288, *Megamonas funiformis* An805, *Megasphaera stantonii* An771, *Megasphaera elsdenii* An838, *Phascolarctobacterium* sp. ET69, *Bifidobacterium saeculare* An816, *Succinatimonas hippei* ET63, *Sutterella massiliensis* An829 and *Campylobacter jejuni* NCTC11168 (Table 1). The strains were deliberately selected because they are common in adult hens, usually absent in chicks from hatcheries and capable of efficient caecum colonisation [2,12,24,25]. The strains were grown in Wilkins–Chalgren broth under an anaerobic atmosphere (10% CO_2_, 5% H_2_ and 85% N_2_ atmosphere) at 37 °C for 48 h [12]. Fresh bacterial cultures were used for oral inoculation of three chicks with a volume of 0.1 mL that contained approximately 10^7^ CFU of each strain. For the remaining 2 species, *E. coli* and *Blautia*, 4 chicks naturally colonised by these species were used. These chicks were identified in our previous experiments as accidentally colonised to a high extent by these species according 16S rRNA sequencing, which showed that *E. coli* and *Blautia* formed more than 10% of the total bacterial population. For *E. coli*, publicly available sequence data were utilised. For *Blautia*, DNA originally used as a template for 16S rRNA gene targeted PCR was subjected to shotgun sequencing, contigs belonging to *Blautia* were identified and amino acid sequences from protein-coding genes were determined. Original frozen caecal aliquot samples enriched for *E. coli* and *Blautia* were then used for protein purification, and expressed proteins were identified as in the samples from chickens after oral inoculation with pure cultures.

### 4.4. Purification of DNA from Caecal Contents and 16S rRNA Sequencing

Caecal contents were homogenised in a MagNALyzer (Roche, Basel, Switzerland), and the DNA was extracted using a QIAamp DNA Stool Mini Kit (Qiagen, Hilden, Germany). Sequencing of 16S rRNA genes was used to confirm the colonisation by the strain used for oral inoculation as described [2]. Briefly, the extracted DNA was PCR amplified over the V3/V4 region of 16S rRNA genes using a HotStarTaq Plus MasterMix kit (Qiagen, Hilden, Germany). The resulting PCR products were sequenced using a MiSeq Reagent Kit v3 (600 cycle) and a MiSeq apparatus according to the manufacturer’s instructions (Illumina, San Diego, CA, USA).

### 4.5. Protein Sequence Annotation and Database Construction

Whole-genome sequencing of DNA purified from pure culture was described previously [6]. The nearly complete genome of *Blautia* was determined using metagenomic sequencing of 4 samples in which this bacterium was accidentally enriched. Metagenomic raw reads were trimmed and aligned with bwa v0.7.17-r1188 [45] and samtools v1.10 [46]. Chicken-free reads were assembled with megahit v1.2.9 [47] with meta-sensitive preset and binned with CONCOCT v1.1.0 [48], MaxBin v2.2.7 [49] and MetaBat v2 [50]. Quality of bins was checked with BUSCO v4.1.3 [51] and bins belonging to *Blautia* sp. were identified by sendsketch.sh from BBtools kit. Bins were further refined with MetaWRAP v1.3.2 [52]. Genomic scaffolds of pure cultures or metagenome-assembled *Blautia* sp. were annotated by Prokka v1.14.6 [53] with Prodigal v2.6.3 gene calling [54]. Databases used for annotation included Swiss-Prot and HAMAP (accessed in October 2020). These newly annotated protein sequences were used as local databases for Proteome Discoverer v2.4 (Thermo Fisher Scientific, Waltham, MA, USA).

### 4.6. Analysis of In Vivo Expressed Proteins

Protein purification and mass spectrometry followed the protocol described in our previous study [11]. In brief, caecal contents (50–100 mg) were resuspended in 2 mL of 0.1% polysorbate 80, homogenized and centrifuged for 1 min at 50× *g*. Supernatant was transferred to a new tube and centrifuged at 4000× *g* for 10 min. The pellet was resuspended in 100 μL of 1% SDS and incubated at 100 °C for 1 h. Subsequently, the protein lysate was mixed with 1.5 mL of TRI Reagent and processed according to the manufacturer’s recommendations (MRC). Following trypsin (Promega, Madison, WI, USA) digestion, LC-MS/MS analysis of tryptic peptides was performed using a Dionex UltiMate 3000 RSLC liquid chromatograph connected to an LTQ-Orbitrap Velos Pro hybrid mass spectrometer (Thermo Fisher Scientific, Waltham, MA, USA). Analysis of in vivo expressed proteins was complicated by unequal numbers of detected proteins for different strains. To deal with this issue, peptide-spectrum match (PSM) counts were replaced with ranking, and the protein with the highest PSM counts normalised to protein amino acid length was given a value of 1. Next, we selected the top 25 proteins for each bacterial strain and these were used for the definition of basal biological processes and metabolic pathways. Having defined the most characteristic pathways, additional evidence for their expression was then selectively searched among the remaining proteins. The full list of expressed proteins is available in Table S2.

### 4.7. Scanning Electron Microscopy

Bacterial strains were fixed in 3% glutaraldehyde in Millonig’s phosphate-buffered solution, post-fixed in 2% osmium tetroxide in Millonig’s phosphate-buffered solution, dehydrated in 50, 70, 90, and 100% acetone and dried in hexamethyldisilazane. Then the samples were placed on the carbon tabs attached to the aluminium holder and coated with platinum/palladium (Cressington sputter coater 208 HR). The samples were observed under a scanning electron microscope Hitachi SU 8010 (Hitachi High Technologies, Tokyo, Japan).

### 4.8. Statistical Analysis

Downstream processing and statistical tests were performed in R (version 3.4.0; R Foundation for Statistical Computing, Vienna, Austria). Only proteins identified by at least two peptides and at least one of them being unique were used for subsequent analysis. Differences in in vivo expression of the studied strains were visualized by heatmap and/or principal component analysis using Raup–Crick distances.

## 5. Conclusions

Prediction of in vivo biological functions is key for understanding the role of individual species in complex microbial populations colonising the intestinal tract. However, prediction from genomic sequences may provide misleading information, as in the case of *Megasphaera* in which butyrate, acetate, formate and caproate were predicted as end products of its metabolism [41], while the butyrate pathway clearly dominates in *Megasphaera* colonising the chicken caecum. Knowledge of the expression of metabolic pathways such as carbohydrate and amino acid fermentation, motility and the type VI secretion system is thus important for the gradual understanding of the role of individual gut microbiota members and subsequently of the whole community. Such information can be used for the selection of the most appropriate bacteria for inclusion in defined competitive exclusion products, improving the gut health of newly hatched chickens and reducing the need for therapeutic antibiotic administration.

## Figures and Tables

**Table 1:** List of strains used in this study.

Strain	Genome Size (bp)	NCBI BioSample
*Bacteroides helcogenes* ET71	3,628,556	SAMN27738374
*Bacteroides mediterraneensis* An793	3,528,114	SAMN14913619
*Bacteroides plebeius* ET8	3,540,004	SAMN27738370
*Bacteroides caecigallinarum* An428b	4,041,510	SAMN14913555
*Bacteroides salanitronis* An322	3,432,695	SAMN06473719
*Bacteroides caecicola* ET2	3,347,338	SAMN27738368
*Bacteroides coprophilus* ET5	3,639,507	SAMN27738369
*Mediterranea massiliensis* An502	3,881,690	SAMN14913571
*Marseilla massiliensis* ET9	4,084,489	SAMN27738371
*Megamonas funiformis* An805	2,324,468	SAMN14913626
*Megamonas hypermegale* An288	2,143,661	SAMN06473710
*Megasphaera stantonii* An771	2,568,073	SAMN14913603
*Megasphaera elsdenii* An838	2,417,448	SAMN14913652
*Phascolarctobacterium* sp. ET69	1,885,819	SAMN27738373
*Succinatimonas hippei* ET63	2,389,462	SAMN27738372
*Sutterella massiliensis* An829	2,933,700	SAMN14913648
*Bifidobacterium saeculare* An816	2,057,123	SAMN14913635
*Campylobacter jejuni* NCTC11168	1,641,464	SAMEA3672890
*Escherichia coli* MG1655	4,641,652	NC_000913.3
*Blautia* sp.	3,662,079	Table S3

## Data Availability

The datasets generated during the current study are available in the following repositories: Raw sequencing data have been deposited in GenBank under accession number PRJNA1005821 at https://www.ncbi.nlm.nih.gov/bioproject/?term=PRJNA1005821 accessed on 17 August 2023. The raw mass spectrometry proteomic data have been deposited in the ProteomeXchange repository at https://www.ebi.ac.uk/pride/login (accessed on 12 August 2023) with the dataset identifier PXD044520 using the username reviewer_pxd044520@ebi.ac.uk and password uXtXyRjt.

## Supplementary Materials

The following supporting information can be downloaded at: https://www.mdpi.com/article/10.3390/ijms25126505/s1.
